# The Behavioral Inhibition System/Behavioral Activation System Scales: Measurement Invariance Across Gender in Chinese University Students

**DOI:** 10.3389/fpsyg.2021.681753

**Published:** 2021-12-14

**Authors:** Ming Xu, Jinyu Wang, Zhishuai Jin, Lu Xia, Qiaoping Lian, Sizhu Huyang, Daxing Wu

**Affiliations:** ^1^Medical Psychological Center, The Second Xiangya Hospital, Central South University, Changsha, China; ^2^Medical Psychological Institute, Central South University, Changsha, China; ^3^National Clinical Research Center for Mental Disorders, Changsha, China

**Keywords:** behavioral inhibition, behavioral activation, the BIS/BAS scales, factor structure, measurement invariance, gender

## Abstract

**Objectives:** To identify the optimal factor structure of the behavioral inhibition system/behavioral activation system (BIS/BAS) scales and to examine measurement invariance (MI) of the scales across gender among a sample of Chinese undergraduate students.

**Methods:** Convenience sampling was employed to recruit 1,085 subjects. Participants completed the Chinese version of the BIS/BAS scales. A confirmatory factor analysis (CFA) of competing models was conducted to determine the optimal factor model, followed by a test of MI across gender based on the optimal model.

**Results:** A single-group CFA indicated that the modified four-factor structure fits best in the total sample. Multiple-group CFAs demonstrated that configural invariance, weak invariance, strong invariance, and strict invariance models of the four-factor structure of the BIS/BAS scales were all acceptable.

**Conclusion:** The four-factor structure of the Chinese version of the BIS/BAS scales possesses MI across gender.

## Introduction

The reinforcement sensitivity theory (RST), postulated by Gray (1982, 1987), theorizes that there are two primary mechanisms that regulate and control emotions and behaviors. The behavioral inhibition system (BIS) reacts to punishment, non-reward, and novelty stimuli. The BIS decreases behavioral responses to avoid negative consequences. Activation of the BIS is associated with negative subjective emotions, such as anxiety, fear, sadness, and frustration. Conversely, the behavioral activation system (BAS) responds to reward and non-punishment stimuli. Once activated, the BAS triggers approach behaviors and is associated with the experience of positive emotions, such as excitement, happiness, and hope. According to Gray’s RST, BIS and BAS are described as two separate constructs [i.e., the separate subsystems hypothesis (Pickering, 1997)], suggesting two uncorrelated latent factors in RST instruments. The levels of reward sensitivity and punishment sensitivity of individuals are not correlated to each other because of their independent physiological bases. Since empirical evidence to support the orthogonality of the two systems is limited, the joint subsystem hypothesis postulates that under normal circumstances, BIS and BAS may be interdependent and have a joint influence on behavior (Corr, 2002). Consistent with this hypothesis, BIS and BAS scores were interrelated in community samples (Muris et al., 2005; Bjørnebekk, 2008). In extreme conditions, however, Corr (2002) expected both systems to act independently as separate systems. Consistent with this theoretical expectation, there were indications that BIS and BAS were functionally independent in clinical samples (Vervoort et al., 2010). Gray’s RST presumes stable individual differences in BIS/BAS reactivity to punishment and reinforcement stimuli. Variations in BIS/BAS reactivity are related to differences in anxiety and impulsivity and are considered vulnerability factors for psychopathology (Bijttebier et al., 2009). As such, RST is often employed as a framework to study a broad range of psychopathologies.

Carver and White (1994) developed the BIS/BAS scales that measured the fundamental components of Gray’s theory (Gray, 1982). Up to now, the widely used BIS/BAS scales have been employed in both clinical populations (Claes et al., 2006; Scholten et al., 2006) and healthy individuals (Coplan et al., 2006; Segarra et al., 2007; Jones and Day, 2008). Moreover, the BIS/BAS scales have been used in many countries, such as France (Caci et al., 2007), Poland (Müller and Wytykowska, 2005), Spain (Segarra et al., 2007; Revuelta et al., 2018), and Netherlands (Franken et al., 2005). The scales were shown to possess acceptable reliability and validity in all the above mentioned studies. In addition, previous studies have confirmed that the Chinese version of the BIS/BAS scales has acceptable reliability and validity and can be used to evaluate BIS/BAS reactivity in the Chinese population (Li et al., 2008; Tian et al., 2017).

Carver and White (1994) first proposed a four-factor model for the BIS/BAS scales: BIS, Reward Responsiveness (positive reaction to the occurrence or expectation of reward), Drive (persistent pursuit of goals), and Fun Seeking (a willingness to approach a potential reward event on a whim); with the latter three factors belonging to the BAS scale. The majority of previous studies support this four-factor structure (Franken et al., 2005; Müller and Wytykowska, 2005; Cooper et al., 2007; Demianczyk et al., 2014). However, several studies have provided support for a two-factor model, such as BIS and BAS (Jorm et al., 1998; Yu et al., 2011). van der Linden et al. (2007) argued that a two-factor structure was more suitable than a four-factor structure and that the three BAS scales in the four-factor model assessed the same underlying construct. Similarly, the factor structure of the Chinese version of the BIS/BAS scales was still controversial. In 2008, Li et al. explored the structure of the BIS/BAS scales in the Chinese context with a sample of Chinese university students. Through item analysis, the researchers deleted two items with low discrimination power from the 20-item BIS/BAS scales developed by Carver and White (1994). Then the authors conducted exploratory factor analysis (EFA) and confirmatory factor analysis (CFA), with the results finding that a four-factor model fits best in the Chinese population. In this four-factor structure, item 10 (“When I see an opportunity for something I like, I get excited right away.”) belonged to BAS Fun Seeking, while in Carver and White’s study, item 10 belonged to BAS Reward Responsiveness. Li et al. believed that discrepancies came from the diverse understanding of the subjects of the items caused by cultural differences (Li et al., 2008). In Tian et al. (2017) examined the structure of the Chinese version of the BIS/BAS scales with a sample covering both middle school students and university students, finding that a four-factor model fits best in the Chinese population. The researchers deleted the same two items in the BIS subscale as Li et al. (2008) did. In contrast to the structure in Carver and White’s study (Carver and White, 1994), item 3 (“I’m always willing to try something new if I think it will be fun.”) in BAS Fun Seeking was moved to BAS Reward Responsiveness. Tian et al. attributed the variation to different age ranges of the subjects in the studies and cultural differences between East and West. In summary, although previous studies argued that the four-factor model fits best in the Chinese population, the specific structural compositions of the four-factor measure were slightly different. Therefore, the first purpose of this study was to identify the optimal factor structure of the BIS/BAS scales among our participants from Chinese universities.

Guyer et al. (2009) found gender differences in reward- and punishment-related brain activity. This study explored the activation patterns in the brains of a group of adolescents when they assessed how they expected peers to view them. Different patterns of gender-related activation emerged in several regions, such as the ventral striatum, hippocampus, hypothalamus, and insula, which were previously associated with emotional processing. Moreover, differences in BIS/BAS reactivity were found between genders. Many studies reported that women scored significantly higher than men on BIS reactivity (Carver and White, 1994; Caci et al., 2007; Matton et al., 2013). Regarding the BAS, prior studies found inconsistent gender effects on BAS reactivity in different samples. For example, Carver and White (1994) reported that women scored higher than men on BAS Reward Responsiveness in a sample of college students. Verbeken et al. (2012) found that boys scored marginally higher on BAS Drive compared to girls in Belgium. Since the instruments used were the same in both male and female groups, differences in means might arise from both real differences between genders and limitations of the instruments. In other words, the comparison of means between men and women could be problematic if we do not assess the measurement invariance (MI) of the instrument (Vandenberg and Lance, 2000). Some studies have examined the MI of BIS/BAS scales across genders (Campbell-Sills et al., 2004; Morean et al., 2014; Pagliaccio et al., 2016; Vervoort et al., 2019; Toro et al., 2020). To date, however, no study has verified MI of the Chinese version of the BIS/BAS scales across gender. Thus, the second purpose of the current study was to examine MI of the Chinese version of the BIS/BAS scales across gender so that the scales could be used more confidently, and this particular aim has not been addressed in previous research.

In summary, the current study aimed to identify the optimal factor structure of the BIS/BAS scales and to examine the MI of the scales across gender among a sample of Chinese undergraduate students.

## Materials and Methods

### Participants

Subjects were students from four universities in Changsha, Hunan province, China. Through convenience sampling, a total of 1,105 questionnaires were distributed in December 2019. With the help of the teachers of the participants, two trained psychology students went to the classroom during recess to collect data. After being informed of the purpose of this study and the precautions to take, the participants anonymously completed the questionnaires, and a total of 1,085 valid questionnaires were obtained, with an effective rate of 98.19%. The final sample consisted of 1,085 Chinese undergraduates aged between 16 and 24. There were 265 men (24.42%) with an average age of 18.73 ± 1.05 years and 820 women (75.58%) with an average age of 18.68 ± 1.10 years. There were no significant differences in age between men and women. In terms of education, all participants were in Level 6 of the International Standard Classification of Education (ISCED) 2011 (United Nations Educational, Scientific and Cultural Organization [UNESCO], 2018). Participant ethnicities were all Asian.

The study was approved by the Ethics Committee of the Second Xiangya Hospital of Central South University, and all participants signed a written informed consent form.

### Instruments

In this study, the Chinese version of the BIS/BAS scales was used to measure BIS/BAS reactivity. Carver and White (1994) developed the 20-item BIS/BAS scales, and Li et al. (2008) revised the Chinese version with two items deleted (“Even if something bad is about to happen to me, I rarely experience fear or nervousness” and “I have very few fears compared to my friends”). The deleted items were both reversely scored. Since the scale was translated from English to Chinese, there may be some cultural differences, resulting in the poor performance of these two reverse scoring items in the Chinese context. The revised scales are self-reported questionnaires with 18 items, each scored on a four-point Likert system with 1 = strongly disagree, 2 = disagree, 3 = agree, and 4 = strongly agree. The scale consists of two systems: (1) BIS, i.e., 5 items with a total score ranging from 5 to 20 points, and (2) BAS, i.e., 13 items with a total score ranging from 13 to 52 points. The higher the score, the stronger the effect of the behavioral inhibition/activation system. The BAS consists of three subscales: Reward Responsiveness, Drive, and Fun Seeking. Previous studies suggested that the Chinese version of the BIS/BAS scales had good reliability and validity. In Li et al. (2008) study, Cronbach’s α coefficients for the scales were as follows: total scales = 0.70, BIS = 0.59, BAS Reward Responsiveness = 0.72, BAS Drive = 0.66, and BAS Fun Seeking = 0.55.

### Statistical Analyses

The Kolmogorov-Smirnov test and the Mardia test were used for normality tests. The assessment of the intercorrelation among the variables (i.e., the associations between the items and items, and factors and factors) was conducted using Pearson’s r. Internal consistency was evaluated using Cronbach’s alpha coefficient and McDonald’s omega coefficient.

Confirmatory factor analyses of the two- and four-factor models of the BIS/BAS scales were performed to obtain the optimal factor model for use in Chinese undergraduates by comparing the fit indices. Based on the Kolmogorov-Smirnov test and the Mardia test, the robust maximum likelihood with SE and mean adjustments (MLM) estimator was used to analyze the non-normal data (Satorra and Bentler, 2001). Chi-square was not used as a crucial index with the current sample due to its high sensitivity to larger sample sizes (Cheung and Rensvold, 1999). Therefore, the fitting degree of the models was tested with several other fitting indices: comparative fit index (CFI), Tucker–Lewis index (TLI), root mean square error of approximation (RMSEA), and standardized root mean squared residual (SRMR). Notably, the SRMR was calculated using the unbiased estimator (i.e., SRMR_u_) proposed by Maydeu-Olivares (2017). Comparative fit index and TLI values above 0.90 indicate an acceptable fit to the data (above 0.95 indicate excellent). RMSEA values below 0.08 indicate an overall acceptable fit (below 0.05 indicate a good fit). As for SRMR, values below 0.08 indicate an acceptable fit to the data (Hu and Bentler, 1999; Sun, 2005).

After the determination of the best-fitting model, multiple-group CFAs were performed to test the MI of the BIS/BAS scales across gender. The following four aspects of MI were considered: (1) configural model (Model A) to evaluate whether the factor structures among groups were the same; (2) weak invariance (Model B) to test whether the factor loadings were equal between groups; (3) strong invariance (Model C) to examine whether the intercepts of observable variables were equal between groups; and (4) strict invariance (Model D) to test the equivalence of error variance between groups. The four nested steps were conducted progressively (van de Schoot et al., 2012), and a model with higher constraints was only tested after the invariance of a model with lesser constraints was established. The chi-square difference test was avoided due to its susceptibility to sampling size (Cheung and Rensvold, 1999). The methods of fitting index differences were used to test MI with the *difference in CFI between nested models* [ΔCFI] ≤ 0.01, the *difference in CFI between nested models* [ΔTLI] ≤ 0.01, and the *difference in RMSEA between nested Models* ΔRMSEA ≤ 0.015 as indicators of acceptable invariance (Cheung and Rensvold, 2002; Chen, 2007). If strict invariance was supported, independent sample *t*-tests would be performed to test the gender differences of the different factors.

Confirmatory factor analyses were conducted using Mplus 8.3 (Muthén and Muthén, (1998-2017)), the unbiased SRMR index and its CIs were analyzed with lavaan package version 0.6-8 in R (Rosseel, 2012), while SPSS 26.0 (IBM Corp, 2019) was used for other data analyses. Specifically, McDonald’s omega coefficients were calculated with the OMEGA macro in SPSS (Hayes and Coutts, 2020).

## Results

### Descriptive Statistics

The descriptive statistical analysis results of each item of the BIS/BAS scales are shown in Table 1. The absolute value of skewness of the items ranged from 0.002 to 0.446 (< 2.0), and the absolute value of kurtosis ranged from 0.011 to 1.455 (< 7.0), and therefore, data were considered as moderately non-normal (Curran et al., 1996). According to the Mardia test, standardized multivariate kurtosis (std-MK) = 62.03 > 3, so the data did not conform to a multivariate normal distribution (Bentler, 2006). Correlations of the 18 items of the Chinese version of the BIS/BAS scales are shown in Table 2. All correlations were positive and statistically significant (*p* < 0.01).

**TABLE 1:** Description statistics for the BIS/BAS scales among 1,085 university students in China.

Items	M	SD	Skewness	Kurtosis
1. I go out of my way to get things I want.	2.92	0.585	0.401	1.080
2. When I’m doing well at something, I love to keep at it.	3.15	0.582	0.339	1.170
3. I’m always willing to try something new if I think it will be fun.	3.09	0.578	0.378	1.455
4. When I get something I want, I feel excited and energized.	3.23	0.604	0.376	0.592
5. Criticism or scolding hurts me quite a bit.	2.99	0.636	0.446	0.886
6. When I want something, I usually go all-out to get it.	2.77	0.645	0.138	0.030
7. I will often do things for no other reason than that they might be fun.	2.82	0.626	0.296	0.369
8. If I see a chance to get something I want, I move on it right away.	2.88	0.594	0.303	0.628
9. I feel pretty worried or upset when I think or know somebody is angry at me.	2.95	0.637	0.239	0.260
10. When I see an opportunity for something I like, I get excited right away.	3.01	0.611	0.323	0.750
11. I often act on the spur of the moment.	2.68	0.709	0.002	0.312
12. If I think something unpleasant is going to happen I usually get pretty “worked up.”	2.81	0.651	0.151	0.011
13. When good things happen to me, it affects me strongly.	2.85	0.631	0.133	0.021
14. I feel worried when I think I have done poorly at something.	2.95	0.610	0.315	0.689
15. I crave excitement and new sensations.	2.75	0.695	0.215	0.043
16. When I go after something I use a “no holds barred” approach.	2.61	0.681	0.053	0.270
17. It would excite me to win a contest.	3.13	0.601	0.391	1.065
18. I worry about making mistakes.	3.06	0.604	0.303	0.761

*M, Mean; SD, Standard deviation; BIS, behavioral inhibition system; BAS, behavioral activation system.*

**TABLE 2:** Correlations of the 18 items of the Chinese version of the BIS/BAS scales.

Item	1	2	3	4	5	6	7	8	9	10	11	12	13	14	15	16	17	18
1	1.000																	
2	0.479**	1.000																
3	0.485**	0.595**	1.000															
4	0.418**	0.627**	0.603**	1.000														
5	0.234**	0.375**	0.371**	0.410**	1.000													
6	0.503**	0.367**	0.438**	0.404**	0.289**	1.000												
7	0.254**	0.272**	0.419**	0.313**	0.240**	0.343**	1.000											
8	0.480**	0.399**	0.438**	0.395**	0.241**	0.430**	0.332**	1.000										
9	0.185**	0.306**	0.261**	0.350**	0.496**	0.225**	0.203**	0.212**	1.000									
10	0.416**	0.422**	0.464**	0.529**	0.380**	0.404**	0.331**	0.495**	0.388**	1.000								
11	0.197**	0.177**	0.206**	0.223**	0.187**	0.264**	0.345**	0.244**	0.253**	0.287**	1.000							
12	0.172**	0.284**	0.261**	0.315**	0.429**	0.262**	0.202**	0.235**	0.459**	0.371**	0.358**	1.000						
13	0.268**	0.306**	0.329**	0.372**	0.383**	0.293**	0.234**	0.284**	0.393**	0.410**	0.329**	0.460**	1.000					
14	0.224**	0.356**	0.335**	0.384**	0.506**	0.279**	0.220**	0.265**	0.455**	0.356**	0.277**	0.531**	0.473**	1.000				
15	0.314**	0.275**	0.397**	0.319**	0.141**	0.300**	0.387**	0.321**	0.137**	0.338**	0.303**	0.155**	0.280**	0.228**	1.000			
16	0.438**	0.277**	0.392**	0.324**	0.134**	0.422**	0.239**	0.450**	0.092**	0.364**	0.222**	0.164**	0.232**	0.208**	0.418**	1.000		
17	0.390**	0.421**	0.425**	0.516**	0.353**	0.316**	0.264**	0.348**	0.308**	0.535**	0.208**	0.325**	0.433**	0,364**	0.370**	0.353**	1.000	
18	0.204**	0.321**	0.300**	0.380**	0.521**	0.258**	0.219**	0.218**	0.497**	0.320**	0.247**	0.478**	0.389**	0.563**	0.165**	0.101**	0.390**	1.000

**p < 0.01. BIS, behavioral inhibition system; BAS, behavioral activation system.

### Factor Structures and Internal Consistency

The fits of the two-factor model (BIS and BAS) and the four-factor model (BIS, BAS Reward Responsiveness, BAS Drive, and BAS Fun Seeking) (Li et al., 2008; Tian et al., 2017) were compared. As shown in Table 3, the fitting indices of the four-factor model (Tian et al., 2017) are: χ^2^ (129) = 663.419, *p* < 0.001, CFI = 0.910, TLI = 0.893, RMSEA (90% CI) = 0.062 (0.057, 0.066), and unbiased SRMR (90% CI) = 0.050 (0.045, 0.054). Therefore, this four-factor model was the best fitting model for the current data.

**TABLE 3:** Model fit indices for the competing models tested.

Model	χ^2^ (*df*)	*p*	CFI	TLI	SRMR_u_ (90%CI)	RMSEA (90%CI)
Two-factor model	929.640 (134)	< 0.001	0.865	0.846	0.063 (0.058–0.068)	0.074 (0.070–0.079)
Four-factor model (Li)	712.841 (129)	< 0.001	0.901	0.883	0.051 (0.046–0.055)	0.065 (0.060–0.069)
Four-factor model (Tian)	663.419 (129)	< 0.001	0.910	0.893	0.050 (0.045–0.054)	0.062 (0.057–0.066)
Four-factor model (Tian)*	617.084 (128)	< 0.001	0.917	0.901	0.048 (0.043–0.052)	0.059 (0.055–0.064)

*Items 2 and 4 correlated; df, degree of freedom; CFI, comparative fit index; TLI, Tucker–Lewis Index; SRMR_u_, standardized root mean squared residual (unbiased estimator); RMSEA, root mean square error of approximation; 90% CI, 90% confidence interval.

**FIGURE 1:**
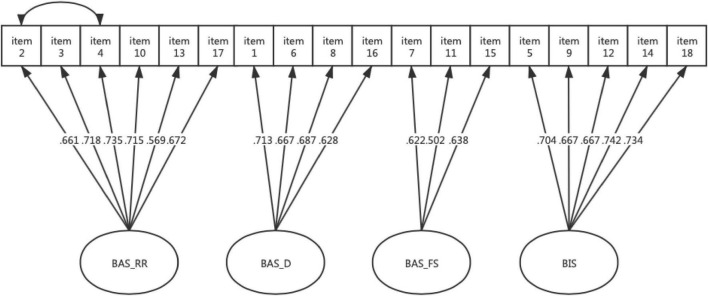
Standardized solution of the modified four-factor structure for the BIS/BAS scales in the Chinese sample. Arrows indicate item factor loadings, which were all statistically significant (*p* < 0.001); the curved line with arrows indicates an error correlation allowed between items 2 and 4. BIS, Behavioral inhibition system; BAS, Behavioral activation system; BAS_RR, BAS Reward Responsiveness; BAS_D, BAS Drive; BAS_FS, BAS Fun Seeking.

Since the initial model was not well fitted to the data (TLI was slightly below 0.9), the model was modified based on both the modification indices reported by Mplus 8.3 and substantive significance to improve the fit of the model, which was consistent with the literature (Yu et al., 2011; Vervoort et al., 2019). In the current study, an error covariance correlation between item 2 (“When I’m doing well at something, I love to keep at it.”) and item 4 (“When I get something I want, I feel excited and energized.”) was allowed. As presented in Figure 1, each item of this modified four-factor model of the BIS/BAS scales has a high loading value on its corresponding factor, ranging from 0.502 to 0.742, which are all statistically significant (*p* < 0.001). The correlations between the four factors ranged from 0.352 to 0.649, which were all positive and statistically significant (*p* < 0.01; see Table 4).

**TABLE 4:** Factor correlations of the modified four-factor structure for the BIS/BAS scales in the Chinese sample.

Factor	BIS	BAS_RR	BAS_D	BAS_FS
BIS	1.00			
BAS_RR	0.621**	1.00		
BAS_D	0.352**	0.649**	1.00	
BAS_FS	0.373**	0.520**	0.499**	1.00

**p < 0.01; BIS, behavioral inhibition system; BAS, behavioral activation system; BAS_RR, BAS Reward Responsiveness; BAS_D, BAS Drive; BAS_FS, BAS Fun Seeking.

Concerning internal consistency, Cronbach’s alpha coefficients and McDonald’s omega coefficients were calculated for the different scales: Cronbach’s alpha was 0.90 for the total scales, 0.83 for BIS (McDonald’s omega = 0.83), 0.88 for BAS, with alpha coefficients for the subscales being 0.84 for BAS Reward Responsiveness (McDonald’s omega = 0.84), 0.77 for BAS Drive (McDonald’s omega = 0.77), and 0.61 for BAS Fun Seeking (McDonald’s omega = 0.61), showing acceptable internal consistency.

Finally, the four-factor model (with one error correlation) was selected as the optimal baseline model for follow-up MI testing across gender.

### Measurement Invariance Across Gender

First, single-group CFAs were employed to examine the structural validity of the BIS/BAS scales in each gender group. As shown in Table 5, the modified four-factor model of the BIS/BAS scales fits well among both men and women. Subsequently, multiple-group CFAs were performed to test for structural invariance across gender, that is, to test whether the forms or patterns of the latent variables of the scales were the same in men and women. Various parameters were allowed to be freely estimated in the configural invariance test (Model A), and the following fit indices were obtained: χ^2^ (256) = 785.901, *p* < 0.001, CFI = 0.913, TLI = 0.895, RMSEA (90% CI) = 0.062(0.057, 0.067) (see Table 5). While the TLI of Model A was slightly below 0.90, all of the other fitting indices met psychometric requirements, indicating that the configural invariance model was acceptable and could be used as a baseline model for the next step of the analysis. Based on configural equivalence, the factor loadings were set equivalent across gender to test weak invariance (Model B), which showed an acceptable fit (see Table 5). ΔCFI and ΔTLI (Model A vs. Model B) were both less than 0.010, and ΔRMSEA (Model A vs. Model B) was less than 0.008, indicating equivalent factor loading across gender. Strong invariance was tested by setting the measurement intercepts of each observable variable invariant across gender. The model (Model C) showed an acceptable fit, and the ΔCFI, ΔTLI, and ΔRMSEA (Model B vs. Model C) values were also within recommended ranges, establishing strong invariance. Taken together, these results indicate that the observable variable intercepts on the latent constructs were equal across gender. Next, under the premise of strong equivalence, the error invariance equivalence was set. Fitting indices (see in Table 5) indicated that the model (Model D) fits well, with ΔCFI, ΔTLI, and ΔRMSEA (Model C vs. Model D) values all meeting fit criteria, supporting the strict invariance across gender. In conclusion, configural invariance, weak invariance, strong invariance, and strict invariance were all established, supporting MI of the BIS/BAS scales across gender.

**TABLE 5:** Fitting indices and model comparisons for measurement invariance models.

Model	χ^2^ (*df*)	*p*	CFI	TLI	RMSEA (90% CI)		Δχ^2^ (Δ *df*)	Δ CFI	Δ TLI	Δ RMSEA
Males	242.694 (128)	< 0.01	0.933	0.920	0.058 (0.047–0.069)		-	-	-	-
Females	533.127 (128)	< 0.01	0.905	0.886	0.062 (0.057–0.068)		-	-	-	-
A	785.901 (256)	< 0.01	0.913	0.895	0.062 (0.057–0.067)		-	-	-	-
B	815.394 (270)	< 0.01	0.910	0.898	0.061 (0.056–0.066)	vs. A	30.384 (14)	0.003	0.003	0.001
C	840.066 (284)	< 0.01	0.908	0.901	0.060 (0.055–0.065)	vs. B	24.672 (14)	0.002	0.003	0.001
D	868.867 (302)	< 0.01	0.906	0.905	0.059 (0.054–0.063)	vs. C	28.801 (18)	0.002	0.004	0.001

*Model A, configural invariance; Model B, metric invariance; Model C, scalar invariance; Model D, strict invariance; df, degrees of freedom; TLI, Tucker–Lewis Index; CFI, comparative fit index; RMSEA, root mean square error of approximation; 90% CI, 90% confidence interval for RMSEA; Δχ^2^, the difference in χ^2^ between nested models; ΔCFI, the difference in CFI between nested models; ΔTLI, the difference in TLI between nested models; ΔRMSEA, the difference in RMSEA between nested Models.*

### Gender Differences in Behavioral Inhibition System/Behavioral Activation System Reactivity

Independent-sample *t*-tests were performed to compare differences across gender in scores on the four-factor model of the BIS/BAS scales (Table 6). Women scored significantly higher

**TABLE 6:** Gender differences in BIS/BAS reactivity among 1,085 university students in China.

	Male (*n* = 265)		Female (*n* = 820)		*t(p)*	Cohen’s *d*
	Mean	SD	Mean	SD		
BIS	14.17	2.646	14.95	2.309	–4.271***(< 0.001)	0.314
BAS_RR	18.04	2.891	18.59	2.600	–2.938** (0.003)	0.212
BAS_D	11.16	2.065	11.20	1.878	–0.283(0.777)	–
BAS_FS	8.17	1.661	8.28	1.476	–0.955(0.340)	–

**p< 0.01, ***p< 0.001; BIS, behavioral inhibition system; BAS, behavioral activation system; BAS_RR, BAS Reward Responsiveness; BAS_D, BAS Drive; BAS_FS, BAS Fun Seeking.

than men on the BIS (*t* = 4.271, *p* < 0.001, Cohen’s *d* = 0.314) and BAS Reward Responsiveness (*t* = 2.938, *p* < 0.01, Cohen’s *d* = 0.212). There were no significant gender differences in the BAS Drive or BAS Fun Seeking factors.

## Discussion

The BIS/BAS scales are widely used to evaluate BIS/BAS reactivity. The current study identified the optimal factor structure and tested MI of the Chinese version of the BIS/BAS scales across gender for the first time in a sample of Chinese university students.

First, results of a single-group CFA showed that the BIS/BAS scales had a four-factor structure in the Chinese sample, which was superior to the two-factor solution. Given the cutoff criteria for the TLI, however, the initial four-factor model proposed by Tian et al. (2017) failed to show an acceptable fitness in the current study, which did not meet the psychometric standards. From the perspective of the data process, we allowed items 2 and 4 error correlation to improve the fit of the model based on the modification indices suggested by Mplus 8.3 and substantive significance, which was similar to previous studies on MI models (Yu et al., 2011; Vervoort et al., 2019; Zhou et al., 2019). Meanwhile, the result of modification has some reasonable and theoretical meanings. Specifically, items 2 and 4 belonged to the same factor (BAS Reward Responsiveness), and it seemed that the two items were more correlated than other items in the factor BAS Reward Responsiveness, which may be caused by similar content and direction. In particular, Yu et al. (2011) added an error covariance correlation between the same items (2 and 4) as the current study. However, we did not delete one of the items because the reliability and validity of the 18-item BIS/BAS scales had been tested in the Chinese context (Li et al., 2008; Tian et al., 2017). If we deleted one of the items, the dimension would be incomplete and would not meet the needs and scientificity of the original scale. The four sub-dimensions: BIS; BAS Reward Responsiveness; BAS Drive; and BAS Fun Seeking, were therefore found to be the best factor model of the BIS/BAS scales, indicating that the four factors of the Chinese version of the BIS/BAS scales were independent which is consistent with previous research results (Li et al., 2008; Tian et al., 2017). The modified four-factor model of the BIS/BAS scales fits well in the total sample and the male and female samples independently, and as such, the four-factor model of the BIS/BAS scales was used as a basic model to study MI of the scales across gender. In this study, BIS and BAS factors were correlated in the sample of Chinese university students, which was consistent with previous studies in community samples (Muris et al., 2005). This finding was in line with Corr’s (2002) joint subsystems hypothesis regarding Gray’s RST, and we need to explore it in clinical samples in the future.

Further multiple-group CFAs showed that configural invariance, weak invariance, strong invariance, and strict invariance of the BIS/BAS scales were all supported, indicating that the BIS/BAS scales possess stability across gender groups. The establishment of configural invariance indicates that the BIS/BAS scales reflect the same psychological structure across gender groups. The determination of weak invariance suggests that there is an equivalent relationship between each item and the corresponding latent variable in gender groups, representing that the scores of the BIS/BAS scales have the same meaning in unit changes in both men and women. Therefore, test scores can be directly compared between men and women. Satisfying scalar invariance suggests that each item on the BIS/BAS scales has the same reference point in men and women. Finally, the establishment of strict equivalence indicates that measurement error caused by random factors is the same across gender. In summary, MI of the BIS/BAS scales across gender was fully established in Chinese university students, supporting that the four-factor structure of the BIS/BAS scales can be used to compare BIS/BAS reactivity between men and women.

With MI between genders supported, the current study compared the scores of men and women on the four factors of the BIS/BAS scales. Analyses found that women scored significantly higher on BIS and BAS Reward Responsiveness than men. Of particular significance, women reported higher BIS reactivity, which was consistent with previous studies (Carver and White, 1994; Caci et al., 2007; Matton et al., 2013). Women showed higher BIS sensitivity, which was consistent with their higher scores on neuroticism (Jorm, 1987; Jorm et al., 1998). Besides, women scored higher on BAS Reward Responsiveness, which was in accordance with previous studies. The idea that BAS Reward Responsiveness possessed a component of neuroticism and negative affectivity on which women tended to score higher than men is an explanation for the gender difference (Jorm et al., 1998; Ross et al., 2002). Since this study has supported the MI of the BIS/BAS scales across gender, the gender differences presented here reflect valid differences in BIS/BAS reactivity levels between genders, rather than inequivalence of the scale itself. What is more, the scales could be used more confidently in China regardless of gender.

The current study has some limitations that should be considered. First, the sample source was limited to university students and therefore generalization of the results to other populations may not be valid. Second, the ratio of men to women in the current study was imbalanced. Future studies should seek to include a more stable ratio. Thirdly, the four-factor model in this study included one error correlation, which, despite its justification, meant that the validity of the scale for the representation of the construct was impaired since the additional variation due to the error correlation contributed to the scores obtained by the measure. Lastly, MI was only tested across gender. It is thus important for future studies to evaluate the factor structure of the BIS/BAS scales in different representative samples, such as race and age.

## Data Availability Statement

The raw data supporting the conclusions of this article will be made available by the authors, without undue reservation.

## Ethics Statement

The studies involving human participants were reviewed and approved by the Ethics Committee of the Second Xiangya Hospital of Central South University. The patients/participants provided their written informed consent to participate in this study.

## Author Contributions

DW conceived and designed the study. MX performed the analysis and prepared the manuscript. All authors were involved in the study conduction, contributed substantially to its revision, and approved the final manuscript.

## Conflict of Interest

The authors declare that the research was conducted in the absence of any commercial or financial relationships that could be construed as a potential conflict of interest. The reviewer HG declared a shared affiliation with the authors to the handling editor at time of review.

## Publisher’s Note

All claims expressed in this article are solely those of the authors and do not necessarily represent those of their affiliated organizations, or those of the publisher, the editors and the reviewers. Any product that may be evaluated in this article, or claim that may be made by its manufacturer, is not guaranteed or endorsed by the publisher.
